# Prussian Blue Mg—Li Hybrid Batteries

**DOI:** 10.1002/advs.201600044

**Published:** 2016-04-15

**Authors:** Xiaoqi Sun, Victor Duffort, Linda F. Nazar

**Affiliations:** ^1^ Department of ChemistryUniversity of Waterloo200 University Ave WWaterlooOntarioN2L 3G1Canada

**Keywords:** Li ion intercalation, Mg–Li hybrid battery, Mg negative electrode, Prussian blue

## Abstract

The major advantage of Mg batteries relies on their promise of employing an Mg metal negative electrode, which offers much higher energy density compared to graphitic carbon. However, the strong coulombic interaction of Mg^2+^ ions with anions leads to their sluggish diffusion in the solid state, which along with a high desolvation energy, hinders the development of positive electrode materials. To circumvent this limitation, Mg metal negative electrodes can be used in hybrid systems by coupling an Li^+^ insertion cathode through a dual salt electrolyte. Two “high voltage” Prussian blue analogues (average 2.3 V vs Mg/Mg^2+^; 3.0 V vs Li/Li^+^) are investigated as cathode materials and the influence of structural water is shown. Their electrochemical profiles, presenting two voltage plateaus, are explained based on the two unique Fe bonding environments. Structural water has a beneficial impact on the cell voltage. Capacities of 125 mAh g^−1^ are obtained at a current density of 10 mA g^−1^ (≈C/10), while stable performance up to 300 cycles is demonstrated at 200 mA g^−1^ (≈2C). The hybrid cell design is a step toward building a safe and high density energy storage system.

## 1 Introduction

Rechargeable Mg batteries have been recently investigated as candidates for high energy density electrochemical storage systems. Their main asset lies in the possibility of employing an Mg metal negative electrode (“anode”) that is inexpensive, safe for storage and handling, possesses a high volumetric capacity (3833 mAh L^−1^), and provides dendrite‐free deposition.^1^ However, the only Mg full cell that has achieved stable long‐term cycling—which employs a Chevrel phase Mo_6_S_8_ cathode in a Grignard–Lewis acid type electrolyte—also provides a relatively low voltage (1.1 V vs Mg).^2^ Further developments of the Mg battery have not been as successful as Li‐ion batteries. The search for new cathode materials is mainly hindered by the high Mg^2+^ migration barrier in solid state structures,^3^, ^4^ while concerns about the active role of the cathode material surface in Mg^2+^ desolvation have been raised recently.^5^ In addition, materials for the positive electrode (“cathode”) current collectors that have been widely used in Li‐ion batteries or served as the cell case—aluminum or stainless steel—are highly prone to corrosion at low voltage (2 V) in the electrolytes that are favored for use with Mg anodes, such as the all‐phenyl complex (APC, i.e., (PhMgCl)_2_‐AlCl_3_ in tetrahydrofuran (THF)).^6^, ^7^, ^8^

To achieve fast ionic transport in the cathode while maintaining the advantages of an Mg anode, Mg—Li hybrid cells have been proposed.^9^ In these systems, an Mg—Li dual salt electrolyte is used: reversible Li^+^ intercalation takes place in the positive electrode material owing to its superior mobility in solid structures compared to the multivalent Mg^2+^. At the anode, Mg is expected to strip/plate first due to its higher redox potential (−2.37 V vs S.H.E.) compared to Li^+^/Li^0^ (−3.04 V vs S.H.E.); however, a recent study interestingly showed that an Mg rich Mg—Li alloy was produced at a similar voltage to Mg metal, suggesting that co‐deposition occurred in the Mg(BH_4_)_2_‐LiBH_4_ dual salt electrolyte.^10^ Nevertheless, the main benefit of Mg batteries is maintained as long as the electrodeposition is dendrite‐free. Owing to the limited electrochemical window imposed by the cell design (<2 V vs Mg), research on hybrid systems has been carried out using low voltage materials such as Mo_6_S_8_, TiS_2_, TiO_2_, Li_4_Ti_5_O_12_, or FeS_2_.^11^, ^12^, ^13^, ^14^, ^15^, ^16^, ^17^, ^18^ Modification of the cell design, employing molybdenum components on the positive side, increased the electrochemical window of the APC electrolyte above 2.8 V.^19^ In order to exploit this breakthrough, positive electrode materials able to survive highly chlorinated environments are needed.

**Figure 1:**
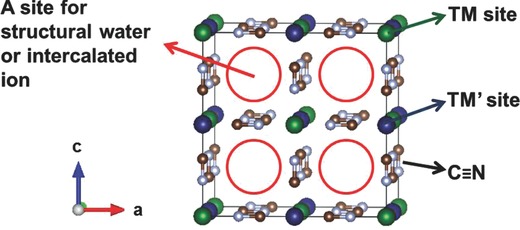
Idealized crystal structure of a Prussian blue analogue (PBA).

Prussian blue analogues (PBA)—ATM[TM′(CN)_6_]_1−_ *_x_*·*y*H_2_O (A = alkali; TM = transition metal)—have recently received attention as cathode materials for rechargeable non‐aqueous Li‐ion,^20^, ^21^, ^22^ Na‐ion,^23^, ^24^, ^25^, ^26^, ^27^, ^28^, ^29^ Ca‐ion,^30^ aqueous divalent‐ion,^31^ and Al‐ion batteries.^32^ In this structure, cyano (C≡N^−^) ligands are highly covalently bonded to the transition metal ions (TM^2+/3+^) owing to strong π‐backbonding, gaining much better chlorination resistance than oxo (O^2−^) ligands. The crystal structure of PBA (**Figure** **1**) is similar to the ReO_3_ and perovskite structure types, being comprised of cubes with TM residing on the corners and bridged by cyano groups along the edges, and thus generating a large central cavity (the A site in the perovskite structure).^33^ Vacancies resulting from the absence of complete [TM′(CN)_6_]^3−/4−^ units (*x* in the above formula), are filled by water molecules with the O atoms occupying the empty sites, and coordinated to the adjacent TM. Water molecules can also be found within the central cavity (A site), either in the exact center of the cavity or slightly shifted due to hydrogen bonding interaction with water molecules coordinated to the transition metal ions.^34^ The A site, at the same time, is also involved in cation intercalation pathways, making the structural water an important factor for the electrochemical properties of PBAs. It has been shown that the transition metal coordinated to carbon has a slightly higher redox voltage in Na cells when structural water is present; however, this water is removed on Na^+^ de‐intercalation.^28^

Herein, we investigate hybrid Mg—Li full cells using either hydrated or vacuum dried PBA (Fe[Fe(CN)_6_]_0.95_·2.3H_2_O and Fe[Fe(CN)_6_]_0.95_·0.7H_2_O, labeled as 23‐PBA and 07‐PBA, respectively) as a cathode material. These are coupled with an APC‐LiCl dual salt electrolyte and an Mg anode, resulting in dendrite‐free deposition on the negative electrode. We demonstrate that PBAs exhibit exceptional corrosion resistance in chlorinated electrolytes such as those typically used in Mg batteries. With an anodic stability of more than 3 V, surpassing even that of the molybdenum current collector, the composition offers reversible capacity at an average voltage of 2.3 V versus Mg in a Grignard‐type electrolyte. We also report the important influence of structural water on cell voltage and capacity retention, and the PBA structural evolution during Li^+^ intercalation.

## 2 Results and Discussion

### 2.1 Materials Characterization—Influence of Water

**Figure 2:**
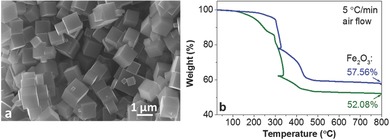
a) SEM image of PBA crystallites; b) TG analysis of 23‐PBA (green) and 07‐PBA (blue).

Both PBA materials are produced in the same morphology, namely, homogenous ≈1 μm cubes as revealed by scanning electron microscopy (SEM) (**Figure** **2**a). Thermogravimetric analysis in air (TGA; Figure 2b) demonstrates that the release of structural water commences around 130 °C, with 07‐PBA losing less mass (5%) than 23‐PBA (14%). The release/combustion of the cyanide group begins at ≈300 °C that yields Fe_2_O_3_ upon completion of the reaction. This allows us to accurately measure the iron content of the sample. In order to avoid inaccuracy arising from the difficulty in precisely deconvoluting the two mass losses, the carbon and nitrogen contents were determined separately by chemical analysis. The remainder of the mass was attributed to water which is in good agreement with the thermogravimetric analysis (Table S1, Supporting Information). The obtained stoichiometries, Fe[Fe(CN)_6_]_0.95_·2.3H_2_O for 23‐PBA and Fe[Fe(CN)_6_]_0.95_·0.7H_2_O for 07‐PBA, show that the vacancy concentration is low and unaffected by the drying process.

**Figure 3:**
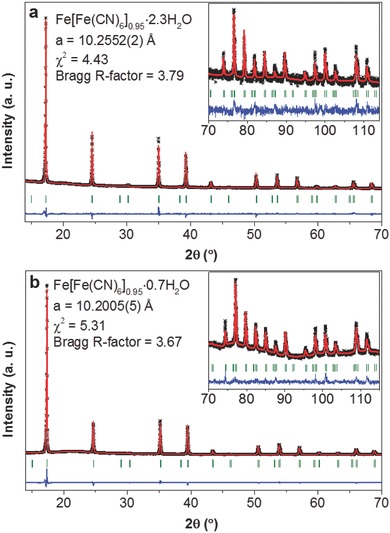
Rietveld refinement results for a) 23‐PBA and b) 07‐PBA (black crosses represent experimental data; red solid lines show fitted data; blue lines show the difference map between observed and calculated data; and green ticks indicate the reflections of the $\mathrm{Fm}\bar{3}m$ PBA phase). Insets show the fit at high angle.

The powder X‐ray diffraction (XRD) patterns of both materials were indexed in the $\mathrm{Fm}\bar{3}m$ (#225) space group, the typical cubic unit cell of PBAs.^33^ Their structures were investigated by Rietveld refinement (**Figure** **3**),^35^ using compositions constrained by the chemical analysis and TGA measurements, i.e., 5% [Fe(CN)_6_]^*n*−^ vacancies, and 14.05 wt% water for 23‐PBA or 4.86 wt% water for 07‐PBA (Table S1, Supporting Information). The empty N site of the [Fe(CN)_6_]^*n*−^ vacancy is occupied by water, fulfilling the octahedral environment of Fe.^34^ The refined structures are reported in Table S2 (Supporting Information). Both H‐bonded (O2, *x,x,x*) and non‐H‐bonded (O3, 0.25,0.25,0.25) water molecules are present in the 23‐PBA structure, whereas only the former exists in 07‐PBA. All of the cavities are occupied by water molecules in 23‐PBA, with 64% H‐bonded and 36% non‐H‐bonded to the coordinated water, whereas only 20% of the cavities are occupied by H‐bonded water in the 07‐PBA structure.

**Figure 4:**
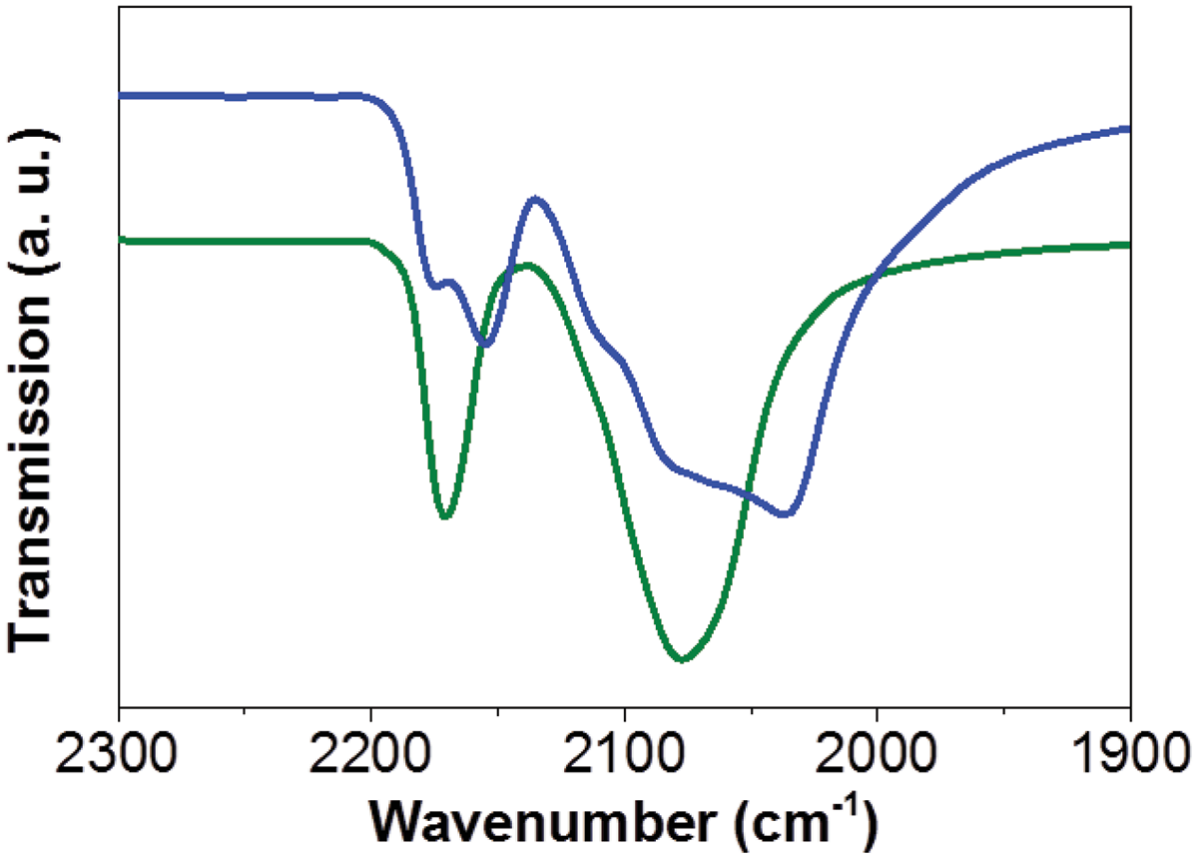
FTIR spectra of 23‐PBA (green) and 07‐PBA (blue), expanded in the CN stretching region.

Removing the structural water results in a shrinkage of the cell parameter from *a* = 10.255 Å in 23‐PBA to *a* = 10.201 Å in 07‐PBA. Analysis of the evolution of the bond distance (**Table** **1**) shows that the contraction is absorbed by the shrinkage of the Fe—C bond. Owing to the hybridization of the π* orbital of the ligand, the shrinkage of this bond implies a weakening of the C≡N bond and should affect the cell voltage. This is in agreement with the Fourier‐transform infrared (FTIR) spectrum of the two PBAs (**Figure** **4**). The peaks at 2080 and 2170 cm^−1^ correspond to the cyanide group with C bonded to Fe^2+^ and Fe^3+^, respectively.^36^, ^37^ The presence of 0.15 Fe^2+^ f.u.^−1^ is expected to compensate for the [Fe(CN)_6_]*^n^* ^−^ vacancies; however, the C≡N vibration in ferricyanide (Fe^3+^) gives a much weaker signal than in ferrocyanide (Fe^2+^) at the same concentration.^38^ Without a standard for the bulk material, an accurate estimation of the concentration of each oxidation state is not possible by FTIR. The cyanide bands of 07‐PBA are more complex than those of the hydrated phase. This could either mean a perturbation of the local symmetry or an inhomogeneous chemical environment due to the lower number of water molecules present in the structure. Moreover, the redshift induced by drying is a direct consequence of the elongation of C≡N bond observed by XRD. The lower content of structural water in 07‐PBA is also confirmed by FTIR (Figure S1, Supporting Information).

**Table 1:** Bond lengths in 23‐PBA and 07‐PBAa)

Bond	23‐PBA	07‐PBA
Fe**—**C	1.991(4) Å	1.936(3) Å
Fe**—**N	2.000(3) Å	2.004(3) Å
C**—**N	1.137(6) Å	1.160(5) Å

^a)^Errors are taken strictly from the refinement. Systematic error is not taken into account.

### 2.2 Electrochemistry

APC, ([PhMgCl]_2_‐AlCl_3_) in THF was used as the basis for the electrolyte in our study. In the absence of any additional salt, very low capacities (≈3 mAh g^−1^) are obtained for both 23‐PBA and 07‐PBA (Figure S2, Supporting Information). This shows that Mg^2+^ does not readily intercalate into PBA either with or without the aid of structural water. However, no corrosion is observed up to 3 V versus Mg for a PBA positive electrode supported on molybdenum foil. Therefore, metal hexacyanates exhibit good resistance to chlorine attack, presumably owing to their structure comprised of robust M—CN bonds. In contrast, metals such as stainless steel or aluminum exhibit corrosion because of the poor stability of their oxide‐passivated surfaces.^39^, ^40^, ^41^ The fact that 23‐PBA does not insert Mg^2+^ in APC is in sharp contrast with behavior in aqueous electrolytes.^31^ Similar to what was observed with layered hydrated manganese oxide, sluggish surface reactions are observed in the non‐aqueous electrolyte whereas rapid Mg^2+^ insertion is observed in aqueous electrolytes.^42^, ^43^ This difference points once more at a specific mechanism through which the Mg^2+^ ions are delivered to the surface of the Mo_6_S_8_ cathode material,^5^ suggesting that multivalent ion desolvation must be activated on the surface of the cathode material.

**Figure 5:**
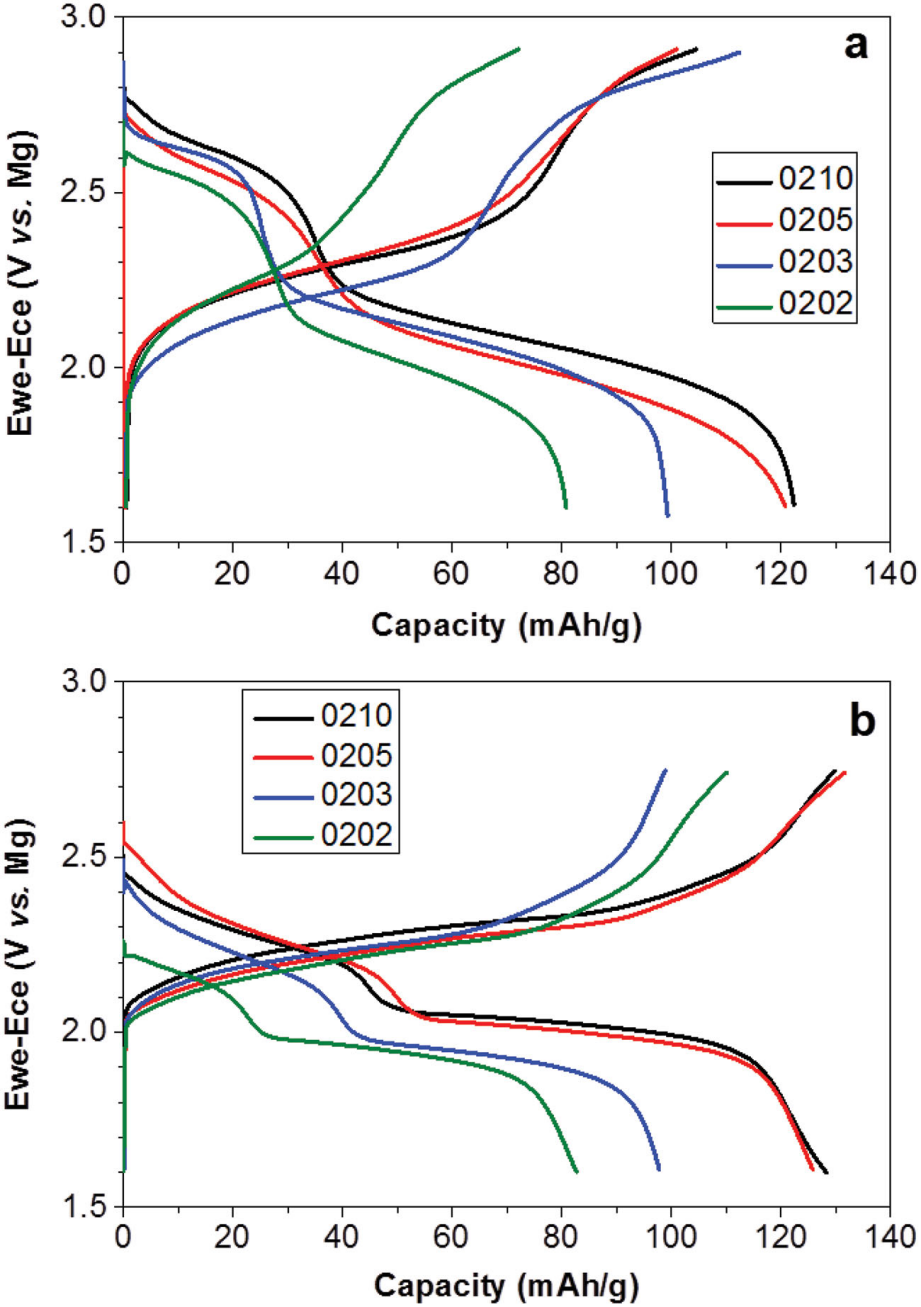
Voltage profiles of a) 23‐PBA and b) 07‐PBA in Mg**—**Li hybrid cells with different salt concentrations (“0205” represents [0.2 m APC + 0.5 m LiCl in THF], as an example) in the electrolyte at a current density of 10 mA g^−1^ (≈C/10) at room temperature. (we = working; ce = counter electrode).

Since Mg^2+^ does not intercalate into the PBA structures, we introduced a monovalent cation, Li^+^, into the electrolyte by adding LiCl. Similar to APC, the dual salt electrolyte also shows reversible metal stripping/plating at the Mg anode and an anodic stability up to 3.2 V with an Mo cathode current collector (Figure S3, Supporting Information). A series of Mg—Li dual salt electrolytes with fixed 0.2 m APC concentration and different LiCl concentrations were examined with both PBA materials in three‐electrode cells^44^ at a current density of 10 mA g^−1^ (≈C/10, where 1C corresponds to a 1 e^−^ transfer per PBA formula). As the LiCl concentration increases up to 0.5 m, the capacity also increases until it reaches the maximum value achieved in this study of 125 mAh g^−1^ for both 23‐PBA (**Figure** **5**a) and 07‐PBA (Figure 5b). Note that considering the mass loading of the positive electrode material and the volume of electrolyte used, about 0.1 m LiCl is required to fill up the PBA structures with the maximum theoretical intercalate content of 1.8 Li per Fe[Fe(CN)_6_]_0.95_·2.3H_2_O or Fe[Fe(CN)_6_]_0.95_·0.7H_2_O (162 and 179 mAh g^−1^). The fivefold lithium excess required to achieve maximum capacity might be due to the complex speciation equilibria of the different ionic species observed in chloride‐based electrolytes,^45^ which yields higher conductivity in electrolyte upon LiCl addition^11^ or traps part of the lithium ions in non‐electrochemically active complexes. Finally, the ±0.1 V overpotential (Figure S4a,b, Supporting Information) for metal stripping and plating in the dual salt electrolyte is similar to what is observed in the pure magnesium APC electrolyte.^6^ The slight deviation among different cells with different LiCl content may arise from unavoidable variation in the sanding procedure used to clean the Mg negative electrode. We note that the plating potential does not reach −0.7 V versus Mg which is normally required to plate Li metal.

**Figure 6:**
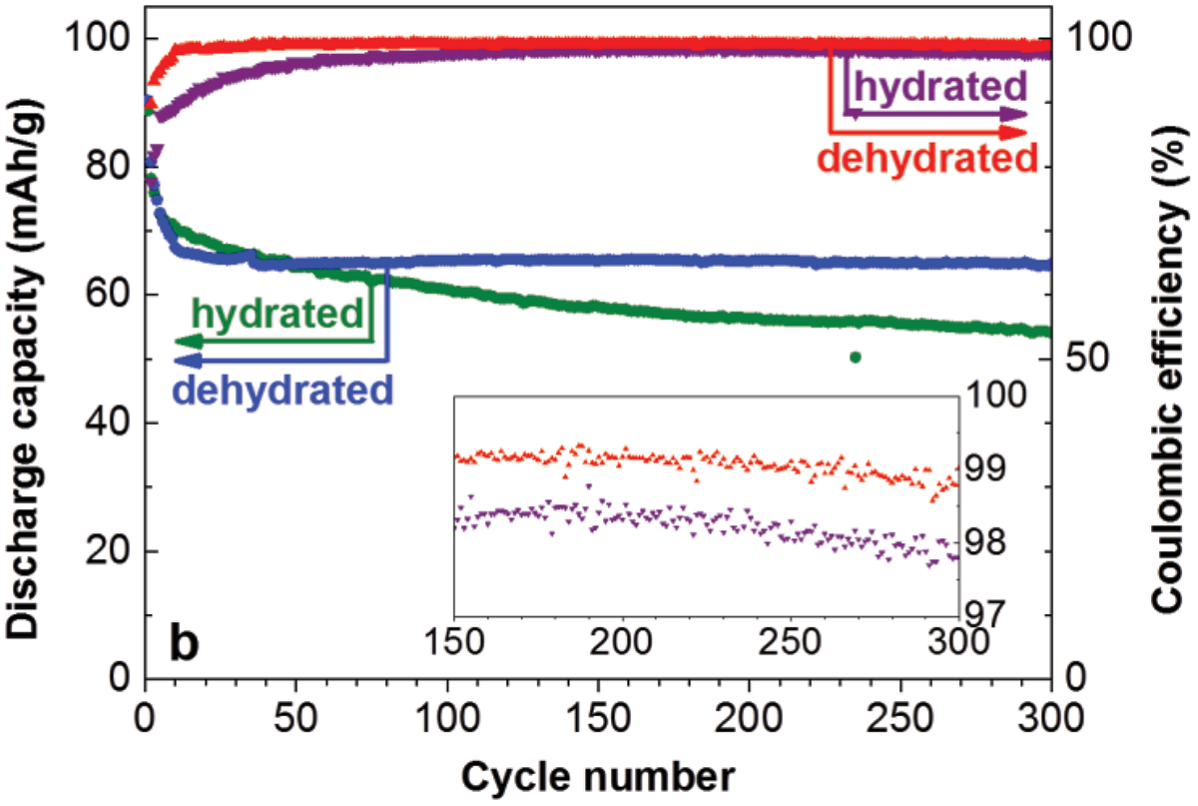
Capacity evolution of 23‐PBA and 07‐PBA in a [0.2 m APC + 0.5 m LiCl in THF] electrolyte. The cells were cycled at 200 mA g^−1^ (≈2C) at room temperature, with a lower voltage cutoff of 1.2 V and charge terminated at 3.2 V (23‐PBA) or 3 V (07‐PBA), the difference in charge cutoff being due to their slighly different average operating voltage.

Long‐term cycling of both materials was studied with [0.2 m APC + 0.5 m LiCl] in coin cells at 200 mA g^−1^ (≈2C, **Figure** **6**). The cell with 07‐PBA shows a drop of capacity in the first ten cycles, and rapidly stabilizes at 65 mAh g^−1^ up to 300 cycles with 99% coulombic efficiency, whereas 23‐PBA displays a slow drop in capacity from 70 mAh g^−1^ at the tenth cycle to 55 mAh g^−1^ at the 300th cycle as well as a slightly lower coulombic efficiency (98%). The higher voltage of 23‐PBA would allow for higher energy density; however, not all Li^+^ could be extracted simply due to electrolyte anodic decomposition at the end of charge. Although structural water remains in 23‐PBA during the first cycle, it does not persist until the end of 300 cycles according to FTIR (see next section). This release of structural water into the electrolyte on long‐term cycling also affects the overall cell performance.

### 2.3 Cathode Insertion Mechanism

**Figure 7:**
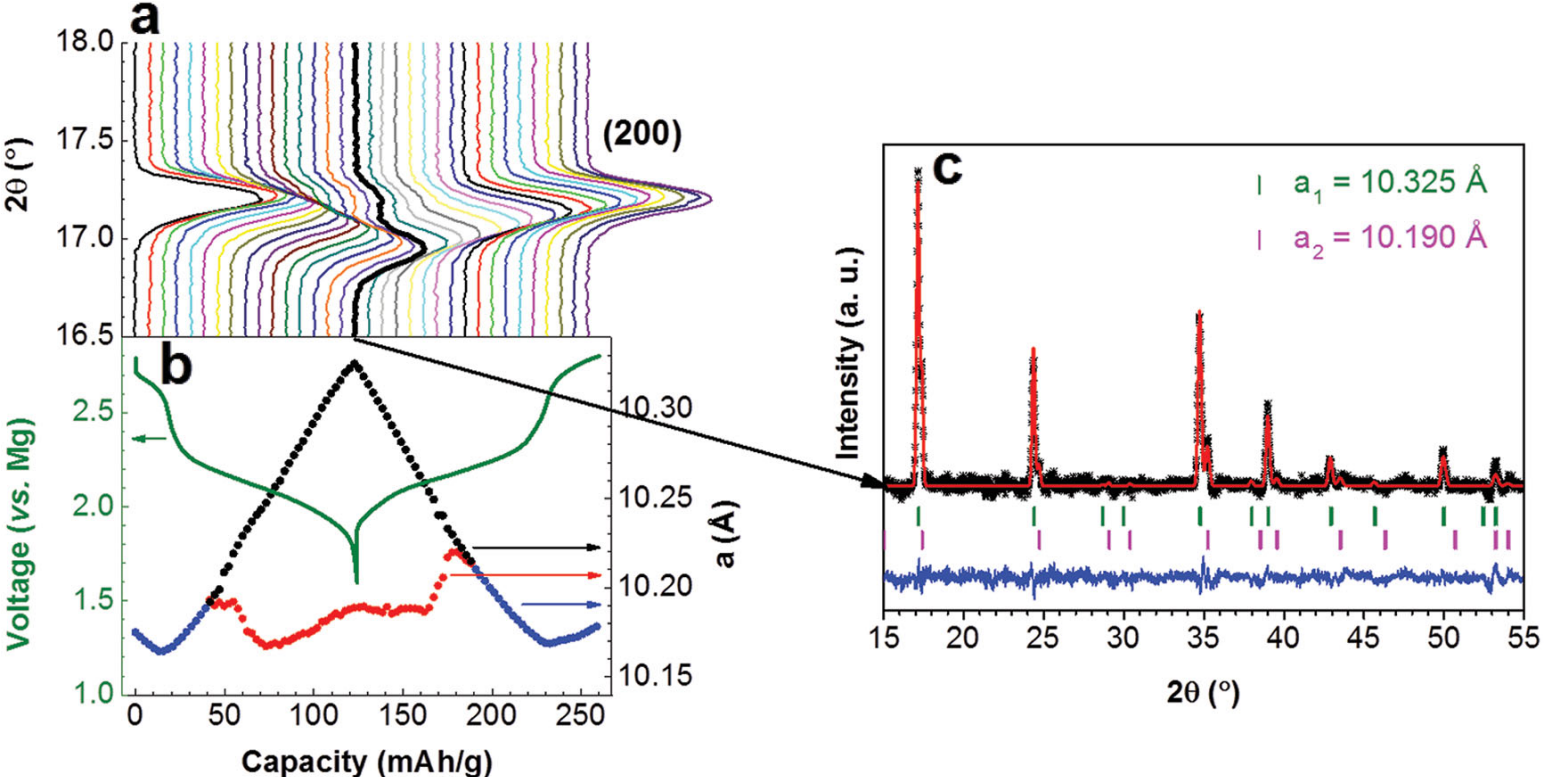
Diffraction data for 23‐PBA cycled in an electrolyte comprised of [0.2 m APC + 0.5 m LiCl in THF] at a current density of 10 mA g^−1^ at room temperature. a) Operando XRD patterns showing only the evolution of the (200) peak in the expanded range from 16.5° to 18° 2*θ* for simplicity (the pattern at full discharge is indicated in bold black); b) cell parameter evolution at points along the electrochemical cycle (blue: single phase during the high voltage plateau; black: Li^+^ de/intercalation phase, and red: Li‐poor phase corresponding to phase separation during the low voltage plateau; green: voltage profile). The smaller cell parameter compared to the pristine material results from partially irreversible Li^+^ ion intercalation during the previous three cycles; c) Le Bail fitting showing two cubic phases obtained at the end of discharge (black crosses represent experimental data; red solid lines show fitted data; blue lines show the difference map between observed and calculated data; and green and pink ticks indicate the reflections of the two Fm‐3m phases).

In contrast to the Chevrel phase performance in a dual salt electrolyte where co‐intercalation of Li^+^ and Mg^2+^ readily takes place,^12^ no Mg is observed in the discharged PBA electrodes by energy dispersive X‐ray spectroscopy (EDX) investigation (Figure S5, Supporting Information). Thus all electrochemical capacities represent Li^+^ de/intercalation only, as expected based on the results without LiCl addition (vide supra). Structural evolution of both PBAs during electrochemical cycling was examined by *operando* XRD in an electrolyte comprised of [0.2 m APC + 0.5 m LiCl in THF]. The cubic cell parameter evolution extracted by a Le Bail fit^46^ of the full patterns is summarized in **Figure** **7** along with the electrochemical profile. As the process is similar for both materials, only 23‐PBA is described in detail here (see Figure S6, Supporting Information, for 07‐PBA). The diffraction data are presented here for the fourth cycle, after the formation cycles. The cell parameter decreases upon Li^+^ insertion during the high voltage process, whereas on the low voltage plateau the XRD pattern is clearly indexed with two different cubic phases (Figure 7c and Figure S6c, Supporting Information), one showing very little cell parameter evolution upon Li^+^ insertion and the second presenting a large increase as the lithium content increases. Such behavior is similar to a two‐phase reaction, with a constant Li‐poor phase and an evolving Li‐rich phase. The fraction of the Li‐poor phase continually decreases upon discharge; however not all of it converts at the end, which prevents the material from achieving full capacity (a 1.8 e^−^ transfer with the insertion of 1.8 Li ions). Similar phase separation has been observed during Na^+^ insertion into Fe[Fe(CN)]_6_]_1−_ *_x_*·*y*H_2_O, where part of the material does not uptake Na^+^ at all.^47^ Interestingly, in 23‐PBA, the majority phase exhibits the major change in lattice parameter; conversely, in the case of the dried sample, the majority phase maintains an almost constant cell parameter. This suggests that the phase segregation is due to inhomogeneity in the water content of the cathode material; water‐rich phases being more prone to correlation between intercalate content and cell parameter than a water‐poor phase. The charge process follows a reverse mechanism showing the good reversibility of Li^+^ intercalation.

**Figure 8:**
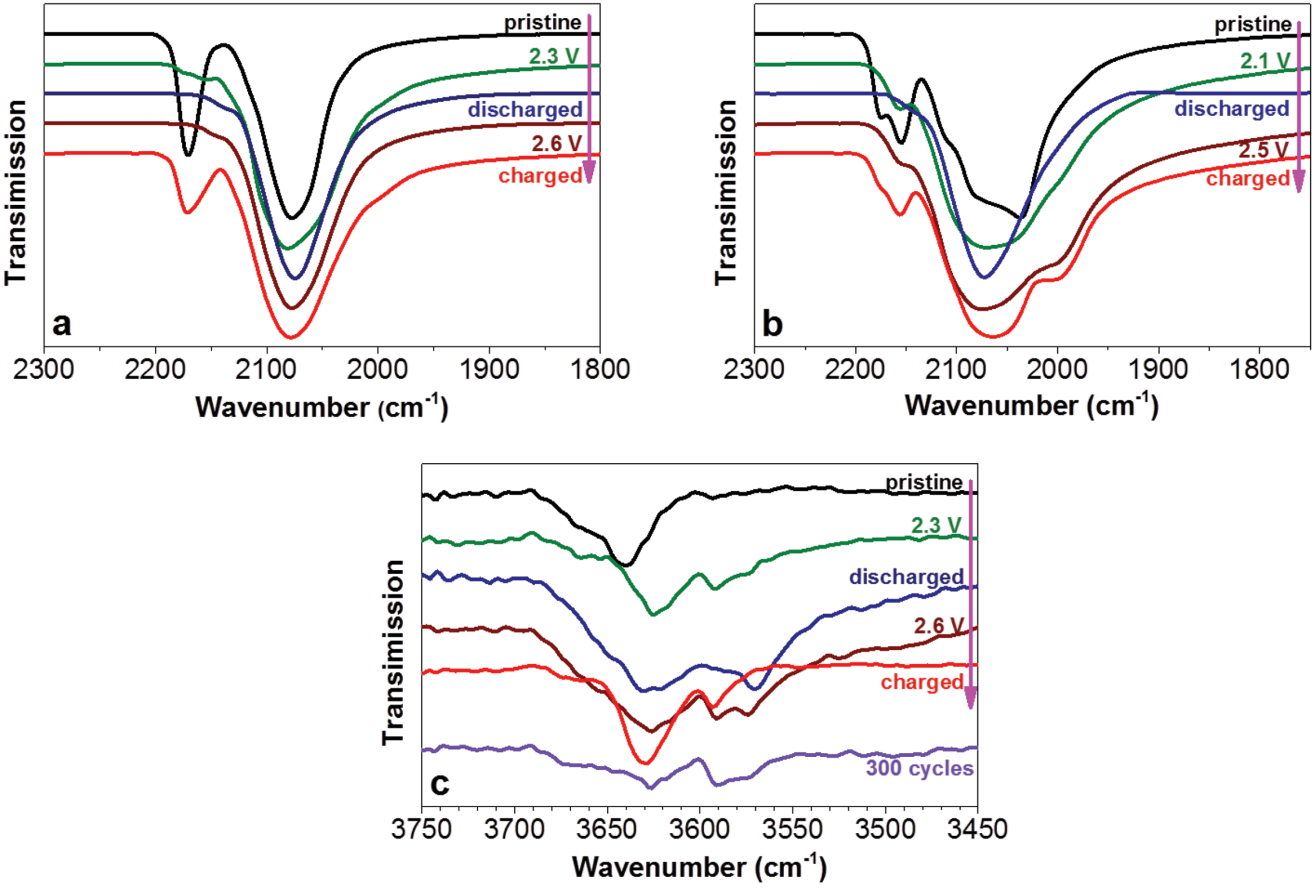
a,b) CN and c) OH stretching band evolution probed by ex situ FTIR spectroscopty on a,c) 23‐PBA and b) 07‐PBA. (Black: pristine; green: end of first discharge plateau; blue: end of second discharge plateau; brown: end of first charge plateau; red: end of second charge plateau; violet: after 300 cycles.)

Two voltage plateaus are observed in both cases due to the existence of two different Fe^2+/3+^ redox centers. The high stabilization of Fe^2+^ coordinated by the carbon atom of the cyano ligands (Figure S7, Supporting Information) is responsible for the significantly higher voltage of the first plateau; as proven by ex situ FTIR (**Figure** **8**a,b) where the peak for the C≡N stretch with C bonded to Fe^3+^ disappears as the voltage drops to 2.1 V. Owing to the strong hybridization of the π^*^ orbitals of the CN ligand, reduction of Fe^3+^ adds an electron to a bonding orbital, and is responsible for the cell parameter contraction observed at the start of discharge. In contrast, reduction of the other metallic center populates nonbonding orbitals and is expected to have little effect on the bond length. The large increase observed in the case of the water‐rich phase is believed to be due to purely steric effects. In addition, the water content also significantly impacts the oxidation potential of the Fe^2+/3+^‐CN centers while having very little influence on the potential of Fe^2+/3+^‐NC (Figure S8, Supporting Information). This is surprising since the shorter Fe—C bond in 07‐PBA, 1.936(3) Å versus 1.991(4) Å (Table 1), generates a higher crystal field stabilization, indicating that the structural water molecules play an active role in the stabilization of Li^+^. Unlike Na^+^ de/intercalation into PBA, where structural water is extracted together with Na^+^ during the first cycle,^28^ Li^+^ extraction here does not modify the water content as evidenced by the sharp crystalline water peak^48^ in the 3550–3700 cm^−1^ region of the discharged 23‐PBA FTIR spectrum (Figure 8c). A slight shift in frequency results from the water interaction with inserted Li ions. Nevertheless, the decrease of peak intensity after 300 cycles (violet curve) indicates water is still slowly released from PBA structure during the repeated Li^+^ de/intercalation process.

### 2.4 Mg Anode

**Figure 9:**
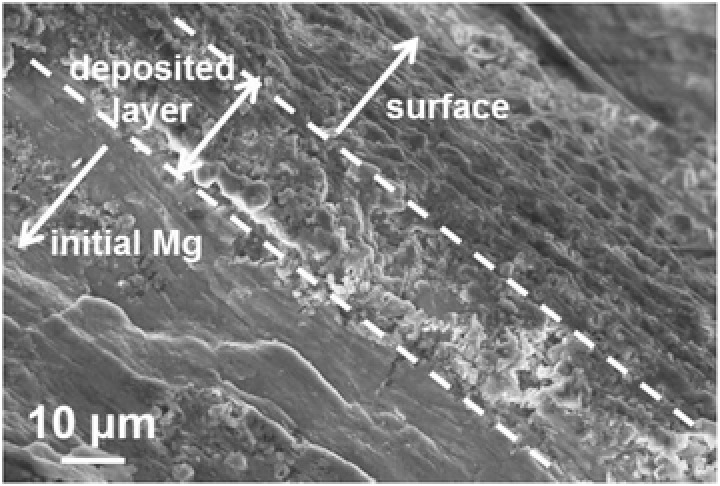
SEM image showing the surface and cross section of the Mg negative electrode after 300 cycles in a hybrid cell at a current density of 200 mA g^−1^.

The morphology of the Mg anode after long‐term cycling was characterized by SEM, showing a dense and dendrite‐free surface (**Figure** **9**) and demonstrating that electrodeposition resembles that of Mg metal rather than Li. Multiple X‐ray photoelectron spectroscopy (XPS) analyses of the electrode surface indicated only the presence of Mg (Figure S9, Supporting Information), suggesting that Li co‐deposition does not occur in this system. Such behavior is expected considering the voltage for metal plating on the anode is at −0.1 V versus Mg (Figure S4, Supporting Information), which is 0.6 V above Li^+^/Li^0^. This is different from that of the Mg(BH_4_)_2_‐LiBH_4_ dual salt electrolyte in diglyme where a magnesium‐rich Mg—Li alloy was formed at a potential close to the magnesium metal theoretical deposition potential.^10^ The nature of the electrodeposition process was further investigated at high rates in Mg/Mg symmetric cells (5 mA cm^−2^), which also results in a non‐dendritic growth (Figure S10, Supporting Information). The stability of the deposited species in the atmosphere was confirmed by placing an electrode strip into water. It was observed to react very slowly, unlike Li metal. Further investigation of the deposition species is not the focus of this work; nevertheless, we show that the primary advantages of using an Mg negative electrode—including dendrite‐free deposition and quasi‐stability in ambient atmosphere—are preserved in this hybrid cell.

## 3 Conclusions

Examination of the Li^+^ insertion mechanism into two iron Prussian blue analogue cathode materials with different water contents—23‐PBA and 07‐PBA—shows that the electrochemical profile, consisting of two voltage plateaus, can be explained based on the basis of two unique Fe bonding environments. The full cell with an Mg counter electrode reaches ≈125 mAh g^−1^ capacity at an average voltage of 2.3 V with a moderate 10 mA g^−1^ rate (≈C/10), resulting in an energy density of 290 Wh kg^−1^. Such results represent higher performance compared to the state‐of‐the‐art hybrid cell with a Chevrel Mo_6_S_8_ cathode that operates at an average voltage of 1.4 V and yields 170 Wh kg^−1^ energy density.^12^, ^13^ At much higher current densities (2C rate), the Prussian blue cell still delivers 150 Wh kg^−1^, which is comparable, albeit a little lower, to the Chevrel that is reported to provide an energy density of 165 Wh kg^−1^. The unique combination of a metal negative electrode and a very robust high voltage insertion material offers an alternative to designs based on Mg^2+^ insertion cathodes. Although hybrid cells are limited by the Li^+^ cation content in the electrolyte, and hence present lower volumetric capacity compared to a hypothetical Mg^2+^ ion intercalation battery, they function with greatly extended cycling. The absence of corrosion at high voltage in this system offers very good life span and a capacity fade of less than 0.011% cycle^−1^ over 300 cycles after initial conditioning of the electrode. Given the vast number of PBAs reported in the literature, our findings encouragingly open new doors for implementation of these materials in such cells. Moreover, dendrite‐free metal deposition at the negative electrode is auspicious for the practical use of such devices.

## 4 Experimental Section

*Synthesis*: Na*_x_*Fe[Fe(CN)_6_]_0.94_ was synthesized following a previously reported procedure^25^ and was used as the precursor. De‐sodiation of the precursor material was carried out by stirring the powdered sample in NO_2_BF_4_ (0.25 m, Sigma‐Aldrich, 95%) in acetonitrile (Caledon, 99.9%, dried over molecular sieves) at room temperature for 24 h under inert atmosphere. Complete removal of Na^+^ was confirmed by EDX. 07‐PBA was obtained by heating the material before and after de‐sodiation at 160 °C under vacuum overnight.

*Characterization*: XRD analysis was performed on a PANalytical Empyrean diffractometer with Cu Kα radiation. For *operando* XRD studies, the cathodes were studied in a home‐made in situ cell at a 10 mA g^−1^ current density. Each XRD scan corresponded to a capacity change of 2.5 mAh g^−1^. Rietveld refinements^35^ and Le Bail fits^46^ were performed using the FullProf suite.^49^ SEM images were obtained with a Zeiss Ultra field SEM equipped with an energy dispersive detector (EDX). Carbon and nitrogen contents were measured by combustion analysis on a 4010 Elemental Analyzer. TGA was performed with a TA Instruments SDT Q600 at a 5 °C min^−1^ heating rate under a dry air flow. FTIR spectra were obtained by pressing the ground materials in KBr (Sigma Aldrich, 99%) pellets in Ar‐filled glovebox and recording the spectra in a Bruker Tensor infrared spectrometer under a dry N_2_ flow.

*Electrochemistry*: The positive electrodes were prepared by mixing the as‐prepared materials with Super P and polyvinylidene fluoride (PVDF, Sigma‐Aldrich, average *M*_w_ ≈ 534 000) in a 8:1:1 weight ratio in *N*‐methyl‐2‐pyrrolidone (NMP, Sigma‐Aldrich, 99.5%) and casting the slurry onto carbon paper. The APC electrolyte was synthesized following a reported procedure.^6^ Mg—Li dual salt electrolytes were prepared by adding anhydrous LiCl (Sigma‐Aldrich, 99.998%) into APC at the desired concentrations. Magnesium metal was polished with carbide paper (Mastercraft, 180 grit SiC) and cleaned prior to use. The 2325 type coin cells or three‐electrode cells (DPM Solutions Inc.)^44^ were assembled in an Ar‐filled glovebox, with the cathode side protected by an Mo disc. Galvanostatic studies of all cells were performed on a VMP3 cycler (Biologic) at room temperature.

*Ex Situ XPS Studies on the Cycled Mg Anode*: XPS on cycled Mg anodes [electrodeposition of Mg on Mo at low current (≈50 μA cm^−2^), electrodeposition of Mg on Mg at higher current (≈5 mA cm^−2^), and on the anode after long‐term cycling with PBA] was performed on a Thermo VG Scientific ESCALab 250. Spectra, referenced to adventitious carbon at 285.0 eV, showed no Li signals, only those from Mg.

## Supporting information

As a service to our authors and readers, this journal provides supporting information supplied by the authors. Such materials are peer reviewed and may be re‐organized for online delivery, but are not copy‐edited or typeset. Technical support issues arising from supporting information (other than missing files) should be addressed to the authors.

Supplementary
