# Supplementary material for: Prussian Blue Mg—Li Hybrid Batteries
Source: Adv Sci (Weinh). 2016 Apr 15;3(8):1600044. doi: 10.1002/advs.201600044 (PMC5074312; doi:10.1002/advs.201600044)
Supplement: Supplementary file 1 — Supplementary [file ADVS-3-0j-s001.pdf]

Copyright WILEY-VCH Verlag GmbH & Co. KGaA, 69469 Weinheim, Germany, 2016.

ADVANCED SCIENCE
Open Access

Supporting Information

for *Adv. Sci.*, DOI: 10.1002/advs.201600044

Prussian Blue Mg–Li Hybrid Batteries

*Xiaoqi Sun, Victor Duffort, and Linda F. Nazar**

Copyright WILEY-VCH Verlag GmbH & Co. KGaA, 69469 Weinheim, Germany, 2013.

Supporting Information

Prussian Blue Analogue Mg-Li Hybrid Batteries

*Xiaoqi Sun, Victor Duffort, Linda F. Nazar**

Table S1. Percentages (wt% represents weight percent and at% represents atomic percent) of each element in both PBAs. C wt% and N wt% are obtained from combustion analysis; Fe wt% is calculated from the Fe_2O_3 wt% given by TGA; the remainder of the mass is assigned to H_2O. Chemical formulae are calculated to be $Fe[Fe(CN)_6]_{0.95}\cdot 2.3H_2O$ for 23-PBA and $Fe[Fe(CN)_6]_{0.95}\cdot 0.7H_2O$ for 07-PBA, respectively.

Sample	C	N	Fe	H_2O
23-PBA	22.780 wt%	26.735 wt%	36.430 wt%	14.055 wt%
	36.2 at%	36.4 at%	12.5 at%	14.9 at%
07-PBA	25.345 wt%	29.535 wt%	40.260 wt%	4.860 wt%
	40.5 at%	40.5 at%	13.8 at%	5.2 at%

Table S2a. Atomic coordinates for 23-PBA (χ^2 = 4.43, Bragg R-factor = 3.79).[a)]

Space group = Fm-3m		a = 10.2552(2) Å		formula = $Fe[Fe(CN)_6]_{0.95}\cdot 2.3H_2O$		
Atom	Wyck.	x	y	z	Occ.	B_{iso} (Å^2)
Fe1	4a	0	0	0	1	1.90(1)
Fe2	4b	0.5	0.5	0.5	0.95	1.90(1)
C	24e	0.3059(3)	0	0	0.95	1.90(1)
N	24e	0.1950(2)	0	0	0.95	1.90(1)
O1	24e	0.1950(2)	0	0	0.05	1.90(1)
O2	32f	0.3364(2)	0.3364(2)	0.3364(2)	0.16(0)	1.90(1)
O3	8c	0.25	0.25	0.25	0.36(0)	1.90(1)

Table S2b. Atomic coordinates for 07-PBA (χ^2 = 5.31, Bragg R-factor = 3.67). [a)]

Space group = Fm-3m		a = 10.2005(5) Å		formula = $Fe[Fe(CN)_6]_{0.95}\cdot 0.7H_2O$		
Atom	Wyck.	x	y	z	Occ.	B_{iso} (Å^2)
Fe1	4a	0	0	0	1	2.04(2)
Fe2	4b	0.5	0.5	0.5	0.95	2.04(2)
C	24e	0.3102(2)	0	0	0.95	2.04(2)
N	24e	0.1965(2)	0	0	0.95	2.04(2)
O1	24e	0.1965(2)	0	0	0.05	2.04(2)
O2	32f	0.3109(6)	0.3109(6)	0.3109(6)	0.05(0)	2.04(2)

a) Errors are taken strictly from the refinement. Systematic error is not taken into account.

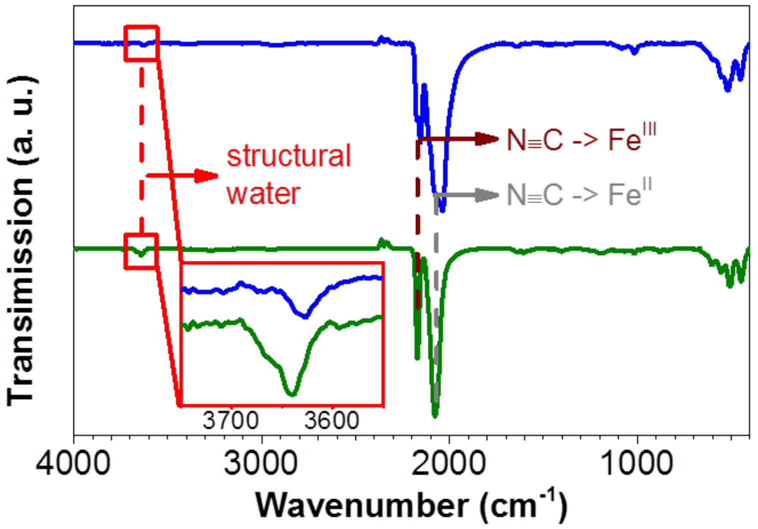

Figure S1. FTIR spectra of 23-PBA (green) and 07-PBA (blue) (inset shows the OH stretch of structural water).

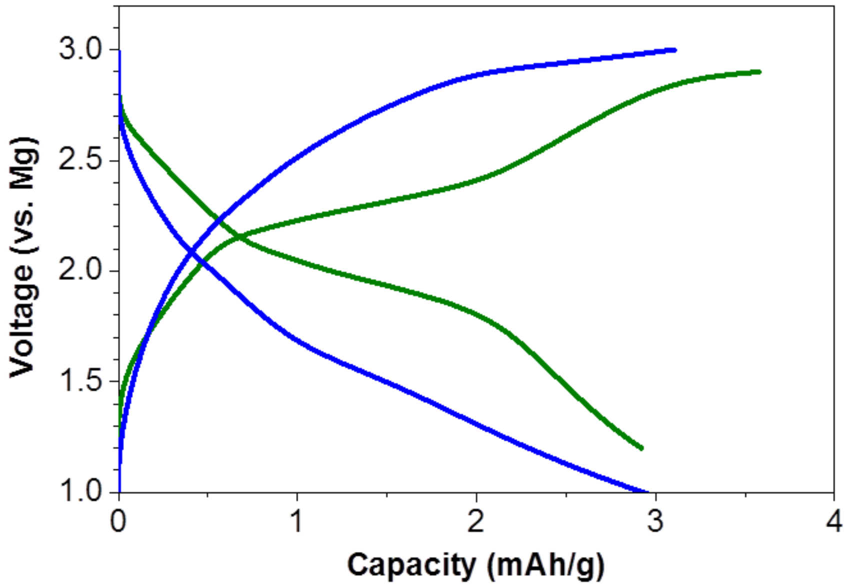

Figure S2. Voltage profiles of 23-PBA (green) and 07-PBA (blue) in APC in THF without Li-salt addition at a current density of 10 mA g^{-1} at room temperature.

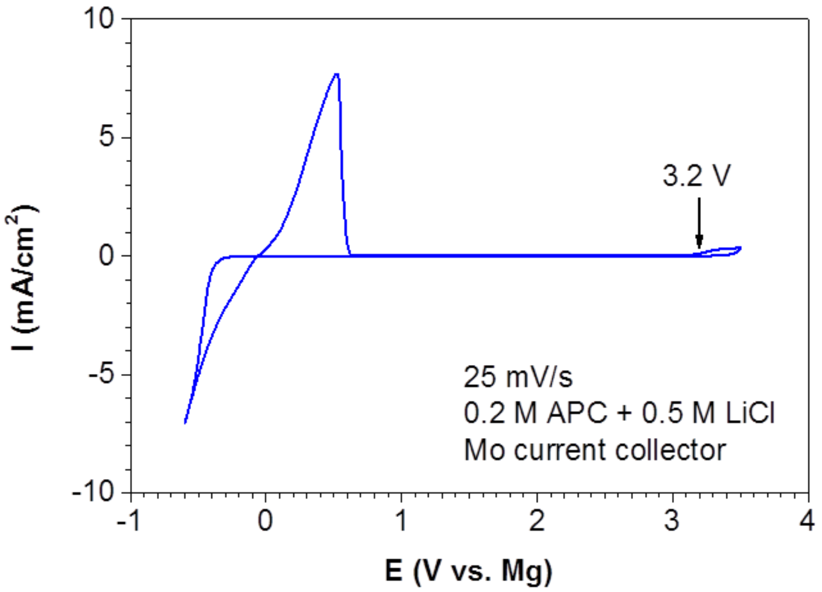

Figure S3. Cyclic voltammogram of the dual salt electrolyte showing reversible metal stripping/plating on Mo current collector and an anodic stability of 3.2V.

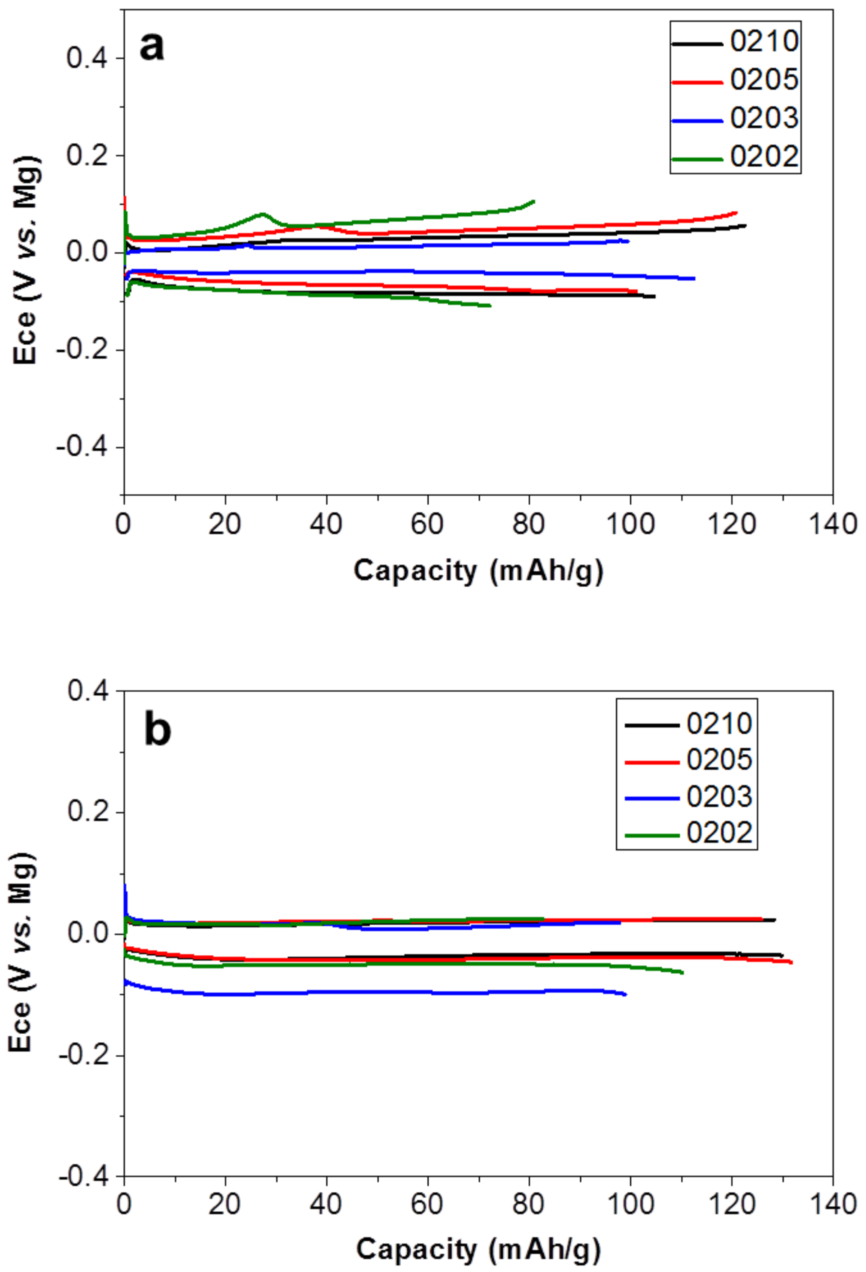

Figure S4. Metal stripping/plating behavior on Mg anode in 3 electrode Mg-Li hybrid cells with (a) 23-PBA and (b) 07-PBA cathode, and different salt concentrations ("0205" represents [0.2 M APC + 0.5 M LiCl in THF], as an example) in the electrolyte at a current density of 10 mA g^{-1} (~ C/10) at room temperature. (ce = counter electrode).

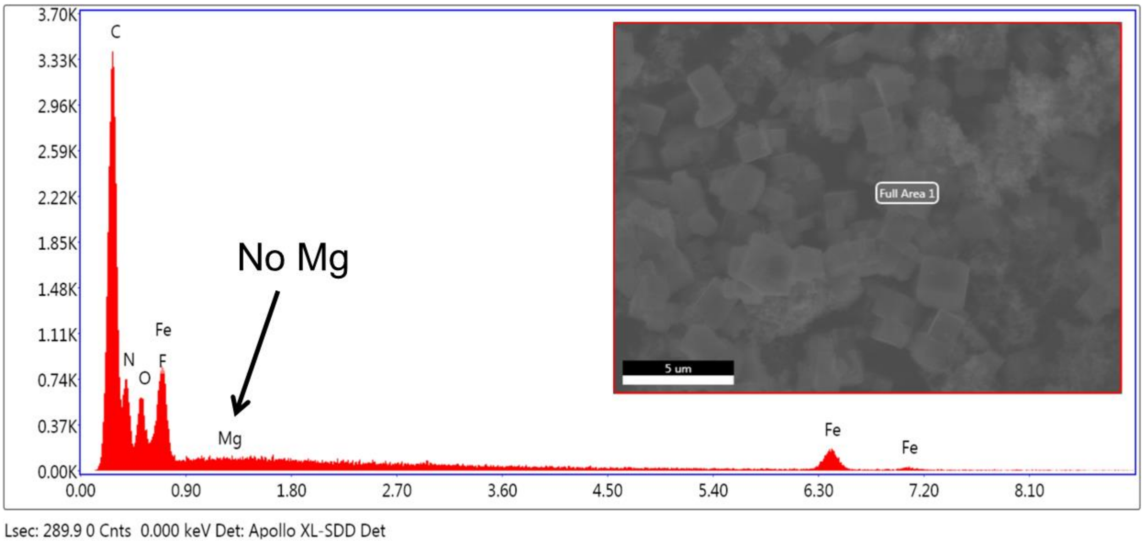

Figure S5. EDX showing the absence of Mg in PBA when discharged in the dual-salt electrolyte (F is due to the PVDF binder).

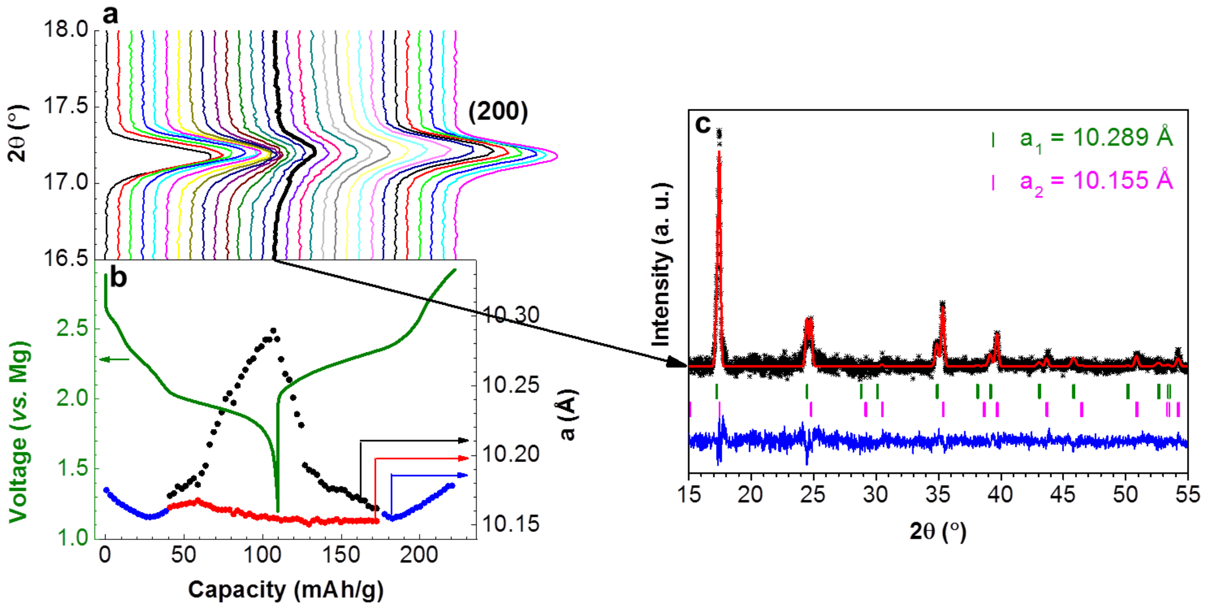

Figure S6. Diffraction data for 07-PBA cycled in an electrolyte comprised of [0.2 M APC + 0.5 M LiCl in THF] at a current density of 10 mA g^{-1} at room temperature. (a) *operando* XRD patterns showing only the evolution of the (200) peak in the expanded range from 16.5 – 18° 2θ for simplicity (the pattern at full discharge is indicated in bold black); (b) cell parameter evolution at points along the electrochemical cycle (blue – single phase during the high voltage plateau; black – Li^+ de/intercalation phase, and red – Li-poor phase corresponding to phase separation during the low voltage plateau; green – voltage profile). The smaller cell parameter compared to the pristine material results from partially irreversible Li^+ ion intercalation during the previous three cycles; (c) Le Bail fitting showing two cubic phases obtained at the end of discharge (black crosses represent experimental data; red solid lines show fitted data; blue lines show the difference map between observed and calculated data; and green and pink ticks indicate the reflections of the two Fm-3m phases).

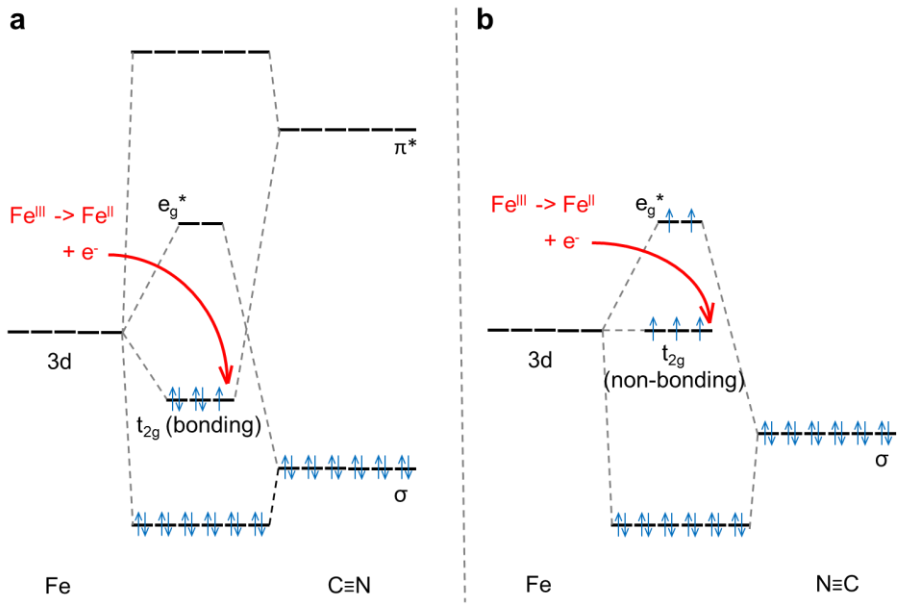

Figure S7. Molecular orbitals of Fe-C≡N (a, -C≡N as π-acceptor) and Fe-N≡C (b, -N≡C as σ-donor)[1]

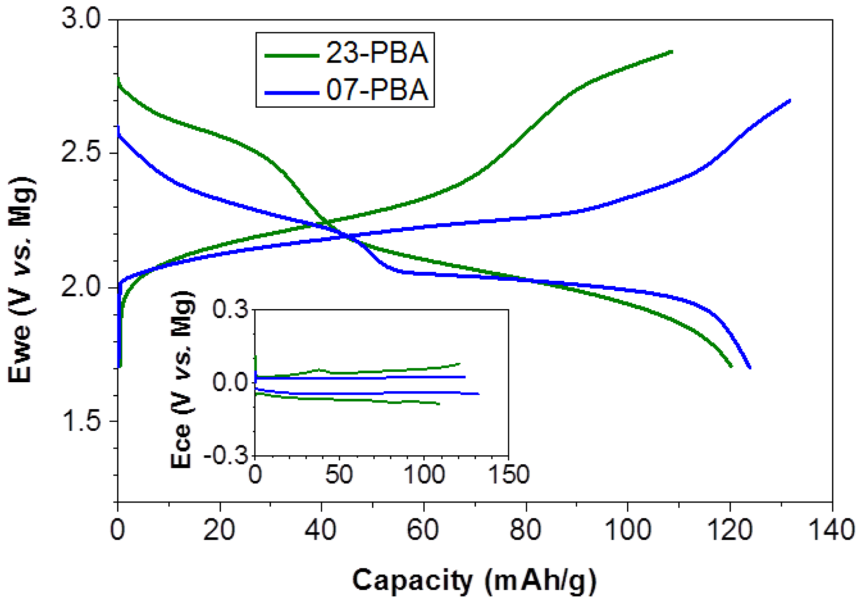

Figure S8. Voltage profile comparison between 23-PBA (green) and 07-PBA (blue) in a [0.2 M APC + 0.5 M LiCl in THF] electrolyte at a current density of 10 mA g^{-1} at room temperature; inset showing similar metal stripping/plating overpotentials on the Mg anode. (we = working electrode; ce = counter electrode).

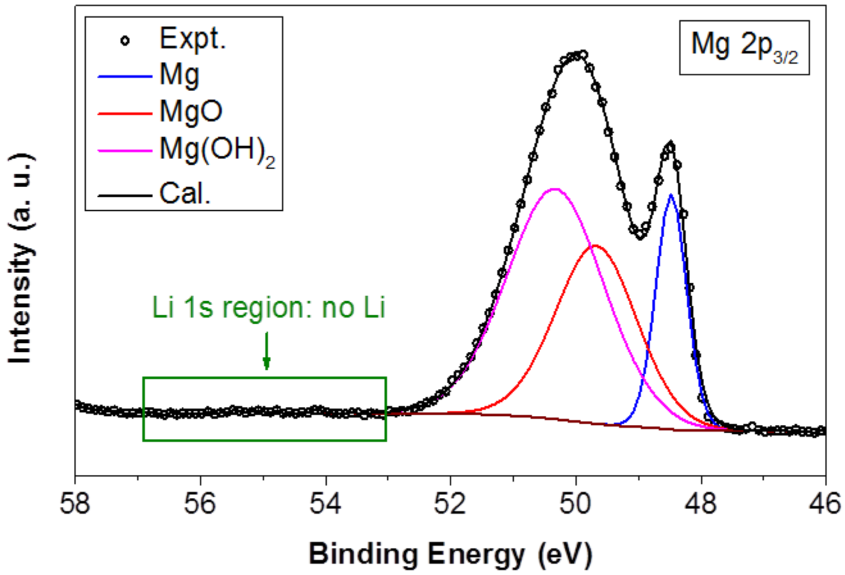

Figure S9. XPS spectrum of the Mg anode after 300 cycles in a hybrid cell at a current density of 200 mA g^{-1}. The absence of a Li signal demonstrates no Li co-deposition occurs.

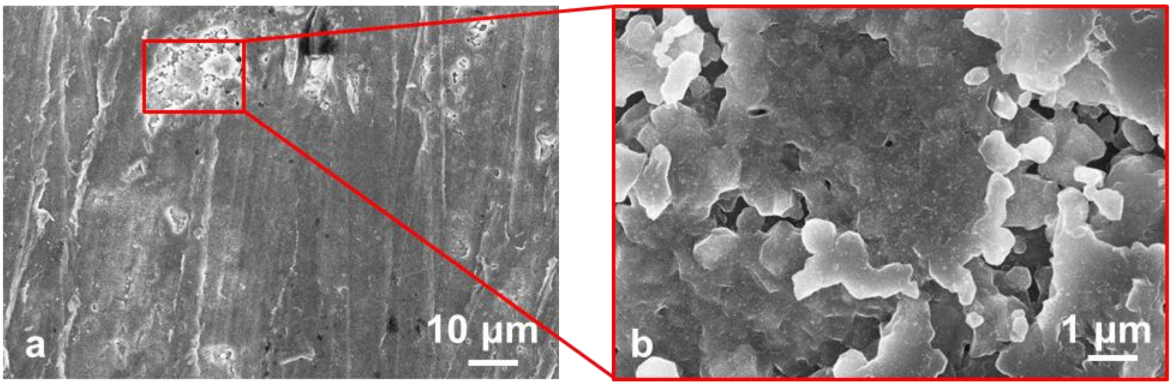

Figure S10. SEM of electrodeposited layer on Mg at 5 mA cm^{-2} current density in a [0.2 M APC + 0.5 M LiCl in THF] electrolyte (the layer was peeled off together with the separator)

References

[1] G. L. Miessler, D. A. Tarr, *Inorganic Chemistry*, 4th Ed, Prentice Hall: Upper Saddle River, NJ, USA **2011**.
